# Incidence, Characteristics, and Outcomes of Interval Breast Cancers Compared With Screening-Detected Breast Cancers

**DOI:** 10.1001/jamanetworkopen.2020.18179

**Published:** 2020-09-25

**Authors:** Saroj Niraula, Natalie Biswanger, PingZhao Hu, Pascal Lambert, Kathleen Decker

**Affiliations:** 1 Section of Medical Oncology and hematology, University of Manitoba, Winnipeg, Manitoba, Canada; 2 Research Institute of Oncology and Hematology, CancerCare Manitoba, Winnipeg, Manitoba, Canada; 3 Cancer Screening program, CancerCare Manitoba, Winnipeg, Manitoba, Canada; 4 Department of Biochemistry and Medical Genetics, University of Manitoba, Winnipeg, Manitoba, Canada; 5 Department of Community Health Sciences, University of Manitoba, Winnipeg, Manitoba, Canada

## Abstract

**Importance:**

Breast cancer comprises a highly heterogeneous group of diseases. Many breast cancers, particularly the more lethal ones, may not satisfy the assumptions about biology and natural history of breast cancer necessary for screening mammography to be effective.

**Objectives:**

To compare tumor characteristics of breast cancers diagnosed within 2 years of a normal screening mammogram (interval breast cancer [IBC]) with those of screen-detected breast cancers (SBC) and to compare breast cancer–specific mortality of IBC with SBC.

**Design, Setting, and Participants:**

In this registry-based cohort study, we collected data about relevant tumor- and patient-related variables on women diagnosed with breast cancer between January 2004 and June 2010 who participated in the population-based screening program in Manitoba, Canada, and those diagnosed with breast cancer outside the screening program in the province. We performed multinomial logistic regression analysis to assess tumor and patient characteristics associated with a diagnosis of IBC compared with SBC. Competing risk analysis was performed to examine risk of death by cancer detection method.

**Exposures:**

Breast cancer diagnosis.

**Main Outcomes and Measures:**

Differences in tumor characteristics and breast cancer–specific mortality.

**Results:**

A total of 69 025 women aged 50 to 64 years had 212 579 screening mammograms during the study period. There were 1687 breast cancer diagnoses (705 SBC, 206 IBC, 275 were noncompliant, and 501 were detected outside the screening program), and 225 deaths (170 breast cancer–specific deaths). Interval cancers were more likely than SBC to be of high grade and estrogen receptor negative (odds ratio [OR], 6.33; 95% CI, 3.73-10.75; *P* < .001; and OR, 2.88; 95% CI, 2.01-4.13; *P* < .001, respectively). After a median follow-up of 7 years, breast cancer–specific mortality was significantly higher for IBC compared with SBC cancers (hazard ratio [HR] 3.55; 95% CI, 2.01-6.28; *P* < .001), for a sojorn time of 2 years. Non–breast cancer mortality was similar between IBC and SBC (HR, 1.33; 95% CI, 0.43-4.15).

**Conclusions and Relevance:**

In this cohort study, interval cancers were highly prevalent in women participating in population screening, represented a worse biology, and had a hazard for breast cancer death more than 3-fold that for SBC. Strategies beyond current mammographic screening practices are needed to reduce incidence, improve detection, and reduce deaths from these potentially lethal breast cancers.

## Key Points

**Question:**

What are the differences and similarities in characteristics and outcomes of breast cancers detected by mammographic screening vs those detected between screening mammograms (interval cancers) in women participating in a population-based screening program?

**Findings:**

In this cohort study of 69 025 women, interval breast cancers accounted for one-fourth of breast cancers in routinely screened women, were 6 times more likely to be grade III, and had 3.5 times increased hazards of breast cancer death compared with screen-detected cancers.

**Meaning:**

Heterogeneity in breast cancer defies assumptions necessary for screening mammography in its current form to be maximally effective; strategies beyond routine screening mammography are needed to prevent, detect, and avert deaths from the more lethal interval breast cancers.

## Teaser

This population-based cohort study examines breast cancer–specific mortality rates associated with interval breast cancer compared with those of screen-detected breast cancers.

## Introduction

Screening mammography, performed every 2 to 3 years, has demonstrated a decrease in breast cancer–specific mortality by 10% to 25% in relative terms, but not overall mortality, compared with no screening.^1^ The concept of mammographic screening stands on 3 primary assumptions: first, breast cancer likely grows in anatomic linearity starting in the breast, then metastasizing to distant organs mostly via regional lymph nodes; second, breast cancers are mostly mammogram-sensitive; and third, frequency of screening is coherent with natural history of breast cancer so that most cancers, particularly the more lethal and/or treatable ones, are detected early by screening.^2^ However, an accumulating wealth of evidence confirms that breast cancer represents a heterogeneous group of highly indolent to fatally aggressive conditions, which presents as a major impediment in the effectiveness of mammographic screening.

Interval breast cancer (IBC) is the cancer detected after a normal screening mammogram but before the next scheduled mammogram. Therefore, IBC by definition defies assumptions necessary for screening mammography to be maximally effective. Few studies have suggested previously that IBCs represent higher grade tumors compared with screen-detected breast cancer (SBC), but the evidence on outcome of IBC compared with SBC is variable.^3,4,5^ When the relationship between method of breast cancer detection and outcome is provided, the effect size is rather small.^6^ In this study, we used a population-based cancer registry to compare the biology and outcomes of SBC with those of IBC.

## Methods

### Data Source and Validity

The study received Manitoba health information privacy committee and University of Manitoba research ethics board approvals. We used the Manitoba Cancer Registry (MCR), the Manitoba BreastCheck registry (provincial registry of screening mammography), and Statistics Canada census data. Written informed consent was waived because the databases were linked using scrambled personal health information number using anonymized versions of each database and all data were deidentified. The MCR, a well validated, robust database was used to identify women aged 50-64 years (age cut-off to have 5 years of follow-up data to determine noncompliant cases) diagnosed with invasive breast cancer from 2004 to 2010, and to collect tumor characteristics. The MCR is a population-based registry that is legally mandated to collect, classify, and maintain accurate, comprehensive information about cancer cases in the province of Manitoba, Canada. The MCR has been shown to be of very high quality including high levels of completeness and histologic verification.^7^

The BreastCheck registry was used to determine the patient characteristics, and screening mammogram date and results. Statistics Canada Census data was used to assess socioeconomic status (average household income) based on neighborhood of residence. Previous studies have shown a high correlation between self-reported household income and a person’s neighborhood average income.^8,9,10^

### Definitions

Screen-detected breast cancer was defined as cancer diagnosed from 0 to 6 months after an abnormal screening mammogram finding; IBC was defined as cancer diagnosed between 0 and 24 months after a normal screening mammogram finding. Women were classified as noncompliant if they were diagnosed with invasive breast cancer more than 2 years after their last screening mammogram. Women who missed at least 1 previous mammogram were regarded as noncompliant. Breast cancer diagnosed in women who did not participate in the screening program were labeled “non–screening program-detected cancers” or cancers detected outside the screening program. Our primary analysis contains the comparison between SBC and IBC. Other groups are reported for exploratory purposes.

### Statistical Analysis

Multinomial logistic regression analysis with age, income quintile, tumor grade, estrogen receptor (ER) receptor, and human epidermal growth factor receptor 2 (*ERBB2*, formerly *HER2*) as covariates was used to assess characteristics associated with a diagnosis of IBC compared with SBC. We adjusted for lead time bias based on Duffy's correction factor,^11^ and performed competing risk analyses using the sub distribution hazard function to examine risk of death by detection method using a sojourn time of 2 years. Sensitivity analyses were done using sojourn times of 1 and 4 years.^12^ Sojourn time is defined as the duration of time when the cancer is in preclinical stage; in other words this denotes the period during which the cancer is detectable by screening mammogram but not clinically.^12^ We investigated the potential for nonlinearity of age in each model, and found age was best modeled as a linear term for all models. The data end point for the survival analysis was June 30, 2010. The date of analysis was March 2020. The *P* value for significance was set at .05. Hypotheses were 2-sided. Analyses were done using SAS statistical software (version 9.4; SAS Institute, Inc). We adhered to the Strengthening the Reporting of Observational Studies in Epidemiology (STROBE) reporting guidelines for reporting observational studies.

## Results

A total of 69 025 women aged 50-64 years had 212 579 screening mammograms from January 2004 to June 2010. There were 1687 diagnoses of invasive breast cancer of which 705 were SBC, 206 were IBC, 275 were noncompliant, and 501 were detected outside the screening program. Tumor and patient-specific characteristics by detection mode are summarized in Table 1. After adjusting for income quintile, ER status, *ERBB2* status, and age, grade III cancers were more likely than grade I cancers to be IBC than SBC (odds ratio [OR], 6.33; 95% CI; 3.73-10.75). Similarly, after adjusting for income quintile, grade, *ERBB2* status, and age, ER-negative cancers had significantly higher odds than ER-positive cancers of being interval compared with screen-detected cancers (OR, 2.88; 95% CI, 2.01-4.13). Mode of cancer detection did not vary significantly by income quintiles or age. Detailed results of multinomial regression analyses including for interval, nonprogram and noncompliant cancers are illustrated in Table 2.

**Table 1.** Summary of Patient and Tumor Characteristics

Detection type	Screening program detected cancer (n = 705)	Interval cancer (n = 206)	Noncompliant cancer (n = 275)	Detected outside screening program (n = 501)	*P* value
Age at diagnosis, mean (SD), y	58 (3. 8)	58 (3.8)	58 (3.6)	58 (3.8)	.06
Stage at diagnosis, No. (%)					
I	446 (63)	51 (25)	126 (46)	149 (30)	<.001
II	207 (29)	92 (45)	99 (36)	189 (38)	
III	45 (6)	49 (24)	39 (14)	104 (21)	
IV	6 (1)	12 (6)	10 (4)	52 (10)	
Unknown	1 (<1)	2 (<1)	1 (<1)	7 (1)	
Income quintile (%)					
1-lowest	97 (14)	28 (14)	49 (18)	82 (16)	.35
2	149 (21)	31 (15)	53 (19)	95 (19)	
3	139 (20)	51 (25)	58 (21)	115 (23)	
4	151 (21)	54 (26)	59 (21)	99 (20)	
5-highest	169 (24)	42 (20)	56 (20)	110 (22)	
Grade					
1	193 (27)	20 (10)	54 (20)	78 (16)	<.001
2	339 (48)	86 (42)	97 (35)	214 (43)	
3	173 (25)	100 (49)	124 (45)	208 (42)	
ER status					
Negative/normal	103 (15)	68 (33)	75 (28)	106 (21)	<.001
Positive/elevated	582 (83)	133 (65)	192 (70)	370 (74)	
Unknown	20 (3)	5 (2)	8 (3)	25 (5)	

Abbreviation: ER, estrogen receptor.

**Table 2.** Results From Multinomial Regression Analysis (Individuals Diagnosed at Ages 52-64 Years)

Covariate	OR (95% CI)		
	Interval cancer	Non–program-detected cancer	Noncompliant cancer
Income quintile			
1 (lowest)	1.16 (0.68-1.99)	1.30 (0.89-1.90)	1.53 (0.97-2.41)
2	0.83 (0.50-1.39)	0.97 (0.69-1.38)	1.07 (0.69-1.65)
3	1.48 (0.93-2.35)	1.27 (0.90-1.79)	1.26 (0.82-1.94)
4	1.44 (0.91-2.28)	1.01 (0.71-1.43)	1.18 (0.77-1.81)
5 (highest)	1 [Reference]	1 [Reference]	1 [Reference]
Grade			
1	1 [Reference]	1 [Reference]	1 [Reference]
2	2.45 (1.46-4.12)	1.57 (1.14-2.14)	1.03 (0.70-1.49)
3	6.33 (3.73-10.75)	2.86 (2.02-4.04)	2.73 (1.84-4.04)
ER			
Positive	1 [Reference]	1 [Reference]	1 [Reference]
Negative	2.88 (2.01-4.13)	1.63 (1.20-2.20)	2.22 (1.58-3.12)
Borderline	0.94 (0.27-3.30)	0.79 (0.32-1.98)	0.65 (0.19-2.30)
*ERBB2*			
Positive	1 [Reference]	1 [Reference]	1 [Reference]
Negative	1.17 (0.36-3.80)	0.62 (0.26-1.45)	2.10 (0.45-9.75)
Borderline	1.10 (0.36-3.35)	1.02 (0.46-2.24)	3.10 (0.70-13.66)
Age			
10-y increase	0.71 (0.47-1.07)	0.69 (0.51-0.93)	0.99 (0.68-1.44)

Abbreviation: OR, odds ratio.

After a median follow-up of 7 years, 170 women had died from breast cancer and 55 women died of other causes. Of the breast cancer deaths, 20 had SBC, 29 had IBC, 27 were noncompliant, and 94 were non–screening program detected. Survival analysis demonstrated that for a sojourn time of 2 years, the unadjusted risk of death from breast cancer was significantly higher for IBC compared with SBC (hazard ratio [HR], 3.55; 95% CI, 2.01-6.28) (Table 3, model 1). Adjusting for income quintile and age at diagnosis did not change the results (model 2). Sensitivity analyses with no sojourn time and sojourn times of 1 and 4 years did not change results significantly, although the effect size decreased with longer sojourn time. Similarly, noncompliant and non–program-detected cancers also had significantly higher hazards of death from breast cancer compared with screen-detected cancers (Table 3). The effect of having a non–screening program cancer on hazards of breast cancer death declined over time (Figure). When adjusted for income quintile and age, non–breast cancer mortality was not increased in IBC compared with SBC cancers (HR, 1.33; 95% CI, 0.43-4.15).

**Table 3.** Risk of Death by Sojourn Time Considering Breast Cancer–Specific Deaths as Event (From 2004-2010 and Invasive Only)

Mode of detection for cancer	No sojourn time		2-year sojourn time	
	HR (95% CI)	*P* value	HR (95% CI)	*P* value
Screening program	1	NA	1	NA
Interval	5.44 (3.08-9.60)	<.001	3.55 (2.01-6.28)	<.001
Non-compliant	3.31 (1.85-5.93)	<.001	2.18 (1.21-3.95)	.002
Non-screening program	10.00 (5.98-16.75)	<.001	6.14 (3.73-10.11)	<.001
Nonscreening program × log(time)	0.52 (0.36-0.74)	<.001	0.56 (0.41-0.76)	<.001
Screening program	1	NA	1	NA
Interval	5.41 (3.06-9.58)	<.001	3.54 (2.00-6.26)	<.001
Noncompliant	3.17 (1.77-5.70)	.001	2.09 (1.15-3.80)	.02
Non–screening program	9.91 (5.92-16.58)	<.001	6.10 (3.70-10.04)	<.001
Non–screening program × log(time)	0.52 (0.36-0.74)	<.001	0.56 (0.41-0.76)	<.001

Abbreviation: NA, not applicable.

**Figure.**
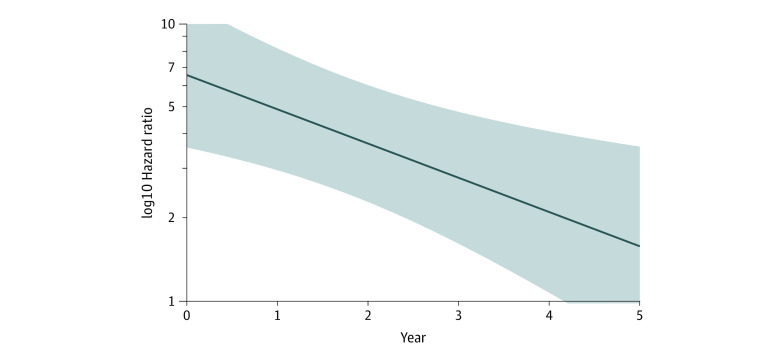
Hazard of Death Over the 4-Year Sojourn Time Hazard of death and 95% CIs for women with non–screening program–detected invasive cancer from 2004 to 2010, changes over the 4-year sojourn time (the solid line indicates the hazard ratio, and the shaded band shows the 95% CIs).

## Discussion

The essence of a cancer screening is its ability to detect target cancer early so that treatments can be offered on time to decrease mortality. Our results suggest that compared with SBC, hazards of death from breast cancer is 3.5 fold higher for IBC in a woman screened under a systematic screening program. Similarly, IBCs were 6 times more likely than SBCs to be of higher grade and about 3 times more likely to be ER negative. A quarter of breast cancers diagnosed in women under such screening program were IBCs.

In line with previous reports, we observed that IBCs are associated with poor tumor characteristics compared with SBCs.^13,14^ Our results shed light on heterogeneity of breast cancer that poses challenges to effectiveness of screening mammography. This could result from 1 or combination of: (a) breast cancers being insensitive to screening mammography; (b) interval between the screening tests (usually 2 years) being longer than the period between origin and development of lethal cancer; (c) false-negative screening mammogram results.

In addition to mass advertisements, every woman in Manitoba is invited via a personal letter to participate in the population-based screening mammography on their 50th birthday. Women diagnosed with either detection modality (IBC and SBC) participated in the screening program, minimizing any systematic population-level differences, although differences owing to selection bias could exist among those who participated in screening vs those who did not, as well as with those who were diagnosed outside the screening program and/or were noncompliant. Data on stage at detection was available but was not included because stage lies in the causal pathway between screening and survival.

### Limitations

The potential for residual confounding by unmeasured or unrecognized factors because of observational data are inherent. Also, although we adjusted for lead-time bias, we did not adjust for length time bias, which could have led to exaggerated survival benefit attributed to screening. Similarly use of cancer-specific mortality does possess the risk of misclassification.

## Conclusions

Our findings challenge previous reports suggesting that IBCs have outcomes similar to SBCs.^3,4,14^ Substantial compromise in outcomes of IBC compared with SBC reflects the differences in natural history of the 2 types of cancers and highlights inadequacies in current breast cancer screening practice. Breast cancer is a highly heterogenous disease; although indolent cancers with likelihood of better outcomes are detected easily by screening mammography raising overall incidence of breast cancer, many of the aggressive and lethal forms of breast cancers either go unnoticed on mammogram or develop in the interval between mammograms. Improvement of breast cancer deaths and overall population mortality requires strategies above and beyond conventional screening mammography. Such strategies could be personalized screening strategies individualizing the screening test based on baseline risks, exploring other methods (eg, tomosynthesis, magnetic resonance imaging), use of artificial intelligence platforms to empower radiology professionals (our group is involved in one), a different frequency of screening, with attention to potential for and consequences of over diagnosis, and be open for reevaluation of population-based screening mammography concept based on risk-benefit ratio in the contemporary context.
